# Morphology-Induced Collective Behaviors: Dynamic Pattern Formation in Water-Floating Elements

**DOI:** 10.1371/journal.pone.0037805

**Published:** 2012-06-08

**Authors:** Kohei Nakajima, Aubery Marchel Tientcheu Ngouabeu, Shuhei Miyashita, Maurice Göldi, Rudolf Marcel Füchslin, Rolf Pfeifer

**Affiliations:** 1 Department of Informatics, University of Zurich, Zurich, Switzerland; 2 Department of Electrical Engineering and Information Technology, Technical University of Munich, Munich, Germany; 3 Department of Mechanical Engineering, Carnegie Mellon University, Pittsburgh, Pennsylvania, United States of America; 4 European Centre For Living Technology, Venice, Italy; Center for Genomic Regulation, Spain

## Abstract

Complex systems involving many interacting elements often organize into patterns. Two types of pattern formation can be distinguished, static and dynamic. Static pattern formation means that the resulting structure constitutes a thermodynamic equilibrium whose pattern formation can be understood in terms of the minimization of free energy, while dynamic pattern formation indicates that the system is permanently dissipating energy and not in equilibrium. In this paper, we report experimental results showing that the morphology of elements plays a significant role in dynamic pattern formation. We prepared three different shapes of elements (circles, squares, and triangles) floating in a water-filled container, in which each of the shapes has two types: active elements that were capable of self-agitation with vibration motors, and passive elements that were mere floating tiles. The system was purely decentralized: that is, elements interacted locally, and subsequently elicited global patterns in a process called self-organized segregation. We showed that, according to the morphology of the selected elements, a different type of segregation occurs. Also, we quantitatively characterized both the local interaction regime and the resulting global behavior for each type of segregation by means of information theoretic quantities, and showed the difference for each case in detail, while offering speculation on the mechanism causing this phenomenon.

## Introduction

Self-organization is one of the ways nature builds artifacts on various scales. Nature offers diverse examples. The formation of molecular crystals [1], the folding of polypeptide chains into proteins [2], the folding of proteins into their functional forms [2], the cells' spontaneous organization into tissues [2], bacteria's organization into colonies [3], [4], or the formation of swarms [5] (such as flocks of birds [6], [7] or schools of fish [8]) at a higher level, are all commonly achieved in a distributed manner, where there is no central control mechanism.

Pattern formation or self-assembling processes can be split into two classes, static and dynamic [9], [10]. Static implies that the resulting structure constitutes a thermodynamic equilibrium. Crystal formation and the assembly of polypeptide chains, for example, can be understood in terms of the minimization of free energy. In the case of biological tissues or swarms of agents, however, one is confronted with systems operating far from equilibrium, which can be classified as dynamic pattern formation. The term “dynamic pattern” indicates that the type of self-organization we are concerned with is observed in a state where the system is permanently dissipating energy and not in equilibrium. In this paper, we focus on dynamic pattern formation and place special emphasis on the role of the morphology of the elements acting on it. “Morphology,” in this context, refers to not only the shape of the elements, but also mechanical properties such as friction coefficients, weight or elasticity.

Investigating self-organization phenomena by focusing on the morphology of system components often provides new perspectives. One prominent example can be found in segregation phenomena, which is defined as a spatial sorting method, where a group of objects occupies a continuous area of the environment that is not occupied by members of any other group, in granular systems [11]. In this context, achieving controlled global segregation behavior has been attracting people's attention. Cohn *et al.* carried out one of the earliest investigations in the beginning of the 1990s [12]. They focused on the role of shape in template and component matching, and studied clustering patterns based on static pattern formation. Followed by several studies on the clustering patterns of passive components, different aggregation patterns with various sizes of colloidal particles were shown [13]. Whitesides *et al.* conducted a series of studies concerning the positional coordination of molecule-mimetic chemistry [14]–[17], reversible aggregation [18], and folding structure [19], [20]. We can find numerous research efforts that have been devoted to the investigation of pattern formation or self-assembling processes in the field. Although these studies suggest that the morphology of elements is important for pattern formation processes, these experiments were mostly performed in static pattern formation. Grzybowski *et al.* conducted a series of studies concerned with dynamic pattern formation [21]–[26]. They prepared millimeter-size magnetized disks or rotors floating at a liquid-air interface and, by rotating an external magnetic field, observed dynamically self-assembled aggregates. In these studies, although the elements assembled only when dissipating energy, the role of the element's morphology in the assembling process was less obvious.

In what follows, we demonstrate simple examples showing that the morphology of the elements can significantly alter the type of segregation in dynamic pattern formation. Our interest is twofold: Firstly, we would like to explore how the control of dynamic pattern formation is embodied in the morphology of the components. This requires an understanding of the underlying interactions and their respective importance for pattern formation. Secondly, our objective is “programming” dynamic pattern formation with an appropriate choice of the morphology. We have deliberately chosen a simple system for our investigations in order to focus on morphology and to study the extent to which system behavior can be altered by morphological means. Our experiments are conducted on the centimeter scale, which consequently enables the observer to track the behavior of individual elements and to directly measure inter-element interactions using simple observation tools (i.e. visual tracking, for example) compared to the experiments on smaller scales. We investigate floating elements on a container filled with water, half of which were actuated by a vibrating buzzer mounted on their tops (active elements), and the other half were merely floating on the water (passive elements). The morphology of the elements is simple: they are either of circular, square, or triangular shape. As long as the system is supplied with energy, we observe specific types of segregation according to each shape. Namely, when the morphology of elements is a circle, the active elements gather in the center of the container and the passive elements are on the periphery. By changing the morphology in the order of circle, square, and triangle, this tendency was gradually inverted, which means that in the triangular elements, the passive elements gather in the center of the container and the active elements were on the periphery. We explain the observed segregation behaviors in detail, and quantitatively characterize each behavior by means of an information theoretic approach. Furthermore, we discuss and speculate on a possible mechanism causing these phenomena, which suggests that the morphology of elements plays a significant role in dynamic pattern formation.

## Results

### Experimental System Overview

**Figure 1:**
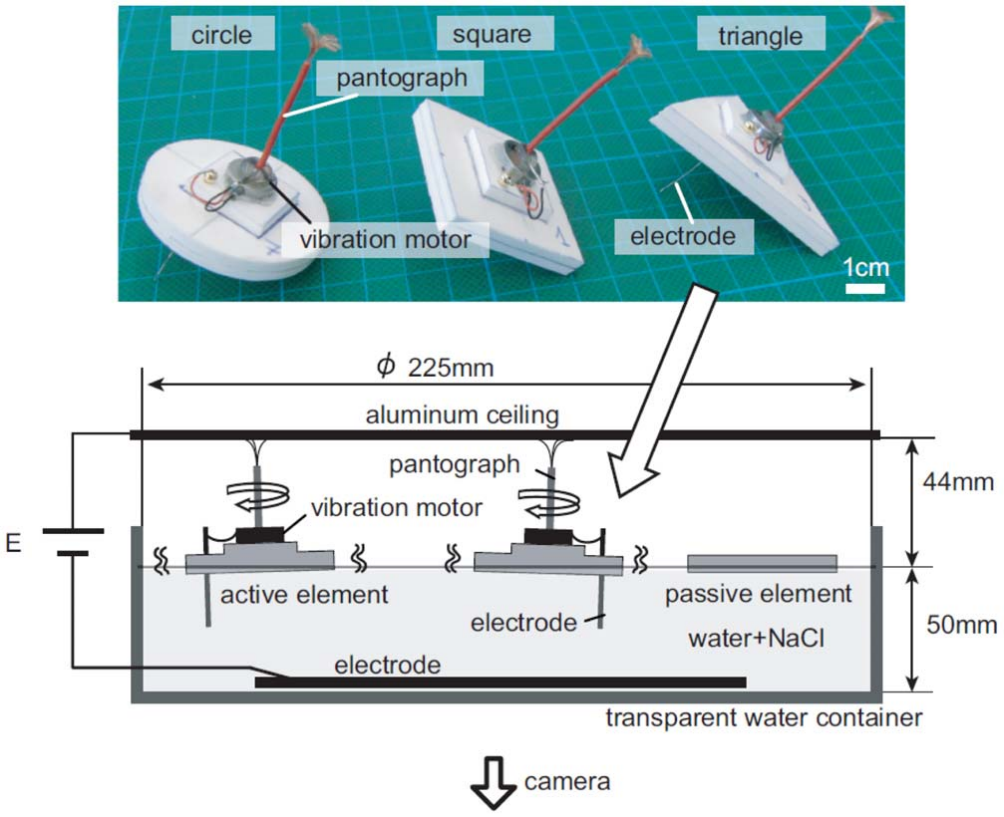
Schematics of experimental setup with three elements. Active elements attain self-agitation by vibrating the vibration motor, while passive elements have no motors.

In order to investigate the effect of morphology on a collection of distributed agents, we employ an experimental platform developed in our group [27]–[32]. The apparatus consists of *cm*-sized floating elements, a water container, and a pantograph system (Fig. 1). For the floating elements, three differently shaped elements were designed (circle, square, and triangle), whose footprints and thicknesses were equally set to 12.25 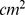 and 6.2 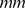, respectively. Each type of element has two different traits, namely active elements and passive elements. The active elements are all equipped with vibration motors that agitate the elements. The passive elements move only when experiencing external forces.

Twelve elements with the same shape were used for each experimental trial. Each set consists of 6 active elements (marked with red) and 6 passive elements (marked with green). The initial configuration of the elements was set as similarly as possible among all shapes (See Materials and Methods section). To start the experiment, we just applied the voltage to the active elements and observed the behavior for 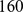 seconds (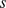) in each trial; the trials were iterated 5 times for the circular, square, and triangular elements. For the analysis of the data, the last 7 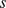 from the total of 160 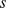 were excluded, and the remaining 153 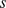 were divided into 2000 timesteps for use in the analysis. Any constraints in the analysis were described in each case. Details on the platform are found in Materials and Methods.

It is emphasized that, in our experimental setting, the movements of the elements were not intentionally controlled. Rather, our preparations solely consisted of setting up the active elements (which vibrated randomly) and the passive elements (which have no motivational force); furthermore, we set the experiment up so there was an equal number of active and passive elements. The behaviors of the elements (or shapes), then, are generated by the interaction between the water-floating elements and the wall of the circular container (see Figure 1). Given the parameters of the rest of our experiment, it can be conjectured that the difference in behavior of the elements is caused mainly by differences in shape.

### Observations

In this section, we explain the observed behavioral differences among the shapes in detail (See Video. S1).

**Figure 2:**
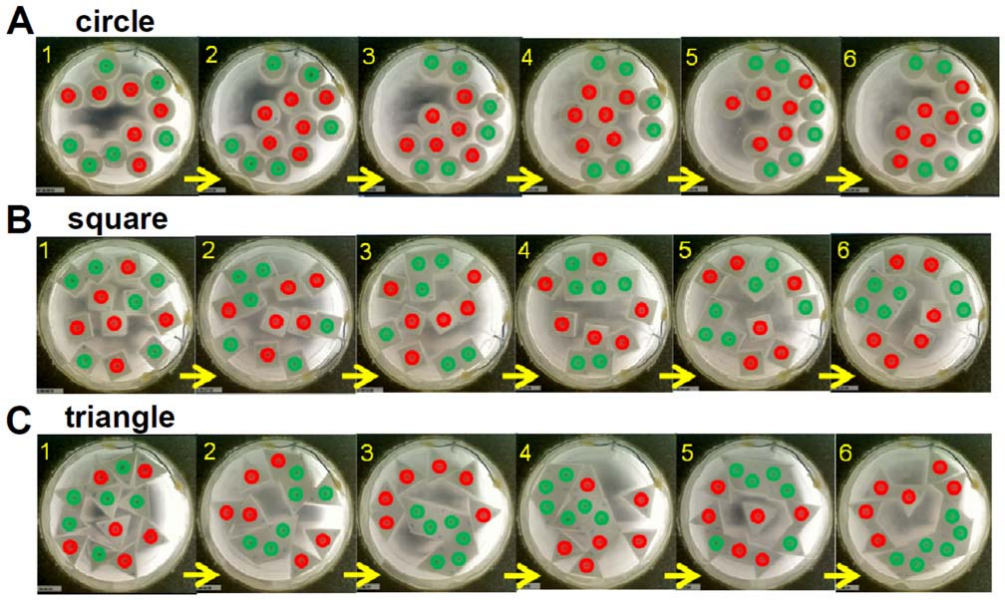
Typical behavior of elements in the circular, square, and triangular cases. **A.** Observation durations of **1.** 8.9 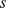, **2.** 13.3 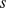, **3.** 52.8 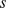, **4.** 77.3 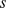, **5.** 112.8 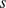, and **6.** 121.2 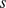 are shown. **B.** Observation durations of **1.** 8.8 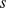, **2.** 27.4 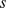, **3.** 53.1 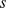, **4.** 93.8 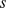, **5.** 125.5 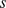, and **6.** 142.2 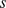 are shown. **C.** Observation durations of **1.** 8.3 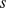, **2.** 44.3 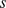, **3.** 56.2 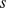, **4.** 69.4 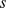, **5.** 93.6 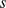, and **6.** 160.0 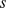 are shown. The colors of the elements are emphasized for clarity. See also Video. S1 for the typical behavior in the circular, square, and triangular cases. The initial configuration of the elements was set as similarly as possible among all shapes (See Materials and Methods section).

In the case of the circular elements (Fig. 2 A), the active elements tended to gather in the center of the container, and the passive elements were pushed to the periphery. This phenomenon, called the *self-organized segregation effect*, remained stable for the entire experiment. The phenomena were also observed in [32] by using circular self-assembling robots with magnetic force. Our results here show that this segregation effect can also happen in the robots without a magnetic force. In what follows, we call this phenomenon “segregation”.

In the case of the square elements (Fig. 2 B), the behaviors were qualitatively different from those in the circular case. The main difference was that several passive elements assembled with each other and formed a kind of cluster (Fig. 2 B2, B3, B4, B5, and B6). In most cases, these clusters consisted of two or three passive elements, or consisted of four or five elements (though this was rare, occurring about two or three times in a single run). These clusters were sometimes pushed to the center of the container by the active elements; the other passive elements that could not form clusters were driven out to the periphery of the container. The active elements were sometimes enclosed at the periphery of the container by the passive elements (Fig. 2 B4). These clusters were not stable and were constantly reconfiguring their formation. Accordingly, the active elements also changed their location, sometimes gathering in the center or at the periphery of the container. The segregation effect between the active and passive elements with a square shape was not as clear as for those with a circular shape.

The triangular elements showed a behavior that was similar to that of the square elements. In particular, the passive elements formed clusters in a fashion similar to those in the case of the square elements (Fig. 2 C2, C3, C4, C5, and C6). One notable difference was that the cluster size (that is, the constituent number of passive elements) was usually larger with the triangles than it was with the squares. We frequently observed clustering of all of the passive elements (Fig. 2 C3, C4, and C6). Segregation was consistently observed with these trials; the cluster of passive elements was pushed to the center of the container, and the active elements remained at the periphery of the container. The cluster of triangles seemed to be more stable than in the square case, but because the shape of the cluster was not a regular polygon; rather, an irregular shape with narrow projections (Fig. 2 C4 and C5), it collided frequently with the active elements, and the reconfiguration of the cluster was observed as well as the square case.

### Trajectory and radial probability distribution in the container

**Figure 3:**
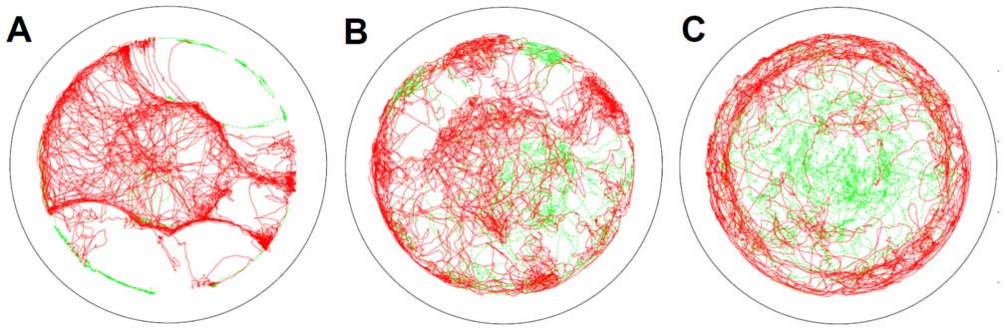
Typical trajectories of the center of the elements in the container. Each figure shows the circular (**A.**), square (**B.**), and triangular (**C.**) cases. The red lines show the trajectories of the active elements, and the green lines show those of the passive elements. Outer circles show the wall of the container. All the trajectories of the elements in one of the trials are overlaid in each figure.

Figure 3 shows the trajectories for each type of element in the container by active and passive shape during an experiment. As observed above, in the case of the circular objects, the trajectories of the active elements are concentrated in the center, and the passive elements stayed at the periphery (Fig. 3 A). The square elements behave differently from the circular elements in that the passive elements are also observed in the center (Fig. 3 B); the triangular elements show this tendency to a greater degree (Fig. 3 C). Accordingly, the active elements are concentrated at the periphery for the triangle.

**Figure 4:**
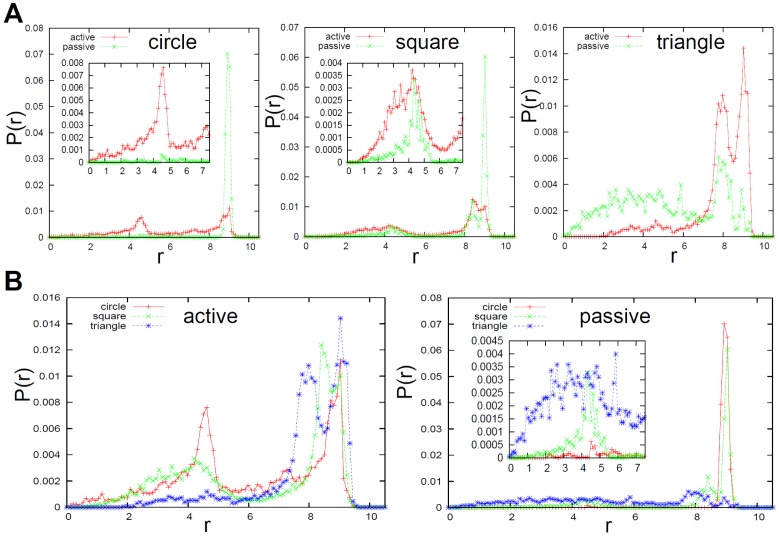
Radial probability distribution of the elements in the container. We calculated the radial probability (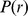) for the active and passive elements according to the distance from the center of the container (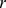; 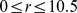). To calculate the probability, we discretized 10.5 *cm* into 100 bins, and used the last 1200 timesteps for all of the trials. **A.** The existence probabilities of the active and passive elements are overlaid for the circular (left), the square (middle), and the triangular (right) element shapes. For the circles and squares in particular, the insets show the enlarged plot of the region, 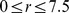, for the same plot. Note that the range of the vertical axis is different for these three graphs. **B.** The existence probabilities of the circular, square, and triangular cases are overlaid for the active (left) and passive (right) elements. Especially in the case of passive elements, the insets show the enlarged plot for the region, 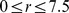, for the same plot. Note that the range of the vertical axis is different for these graphs.

To investigate this segregation feature further, the spatial radial probability (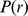) of the passive elements and the active elements in the container was computed according to the distance from the center of the container (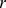 [cm]) (Fig. 4). In Fig. 4 A, we compared the probability distribution between the active elements and the passive elements for each shape. In the circular elements, we can clearly observe that the passive elements concentrate around the periphery of the container (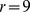), while the active elements concentrate at the center (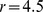) and the periphery (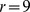) of the container. For the square elements, the structure of the distribution is similar to that of the circular elements. However, the probability of the passive elements being pushed to the periphery is lower than for the circular elements. Furthermore, around the center of the container, it can be seen that the probability of the active elements aggregating at the center is lower, and that this probability is higher for the passive elements when compared with the case of the circular elements. The probabilities for the trajectories of the active and passive elements showing clustering in the center are about the same. In the triangular elements, the structure of the probability distribution is clearly different from that of the other two. We can confirm that the tendency we observed in the case of the square elements is enhanced, so that the probability of the active elements being pushed to the periphery is higher than that of the passive elements, while the probability of the active elements being present in the center is lower than that of the passive elements.

In Fig. 4 B, we can clearly observe that the probability of the active elements around the center of the container decreases when the shape of the elements varies from circle, to square, to triangle in that order. On the other hand, the probability of the passive elements at the periphery of the container also decreases when the shape of the elements varies from circle, to square, to triangle in that order.

To summarize, a similar tendency can be seen in Fig. 4 as in Fig. 3; namely, that the passive elements are more likely to cluster in the center with the triangular elements, and less likely (in descending order) with the square and circular elements. It is clearly seen that the segregation pattern of the active elements and the passive elements gradually reverses according to the shape of the element.

### Traveling distance of elements

**Figure 5:**
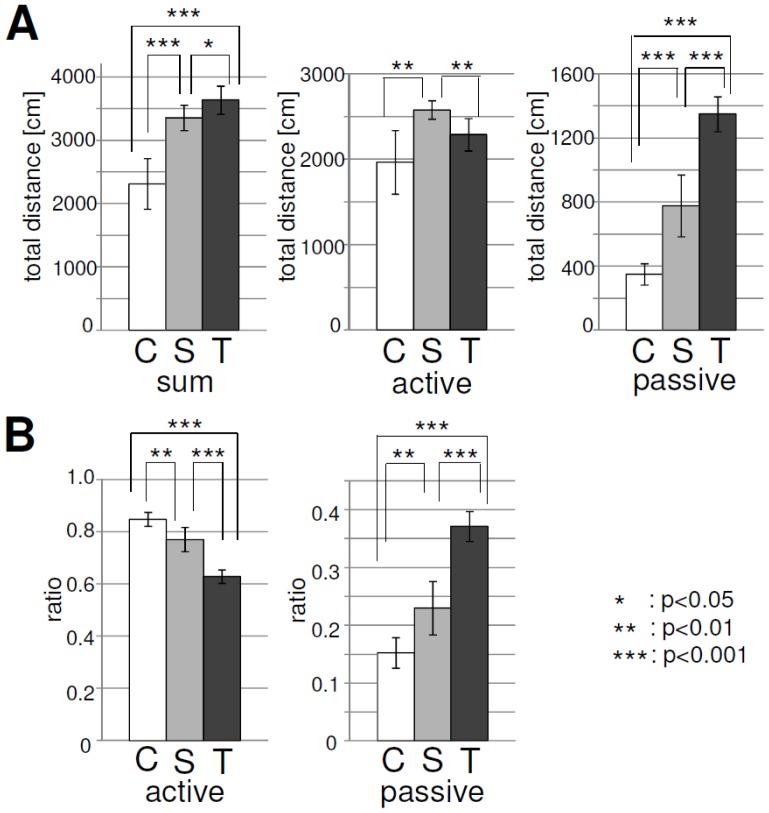
Comparisons of the averaged total traveling distances and the ratio of the traveling distances of the active and passive elements. **A.** Comparisons of the averaged total traveling distances between the circular, square, and triangular elements. Left, middle, and right figures show the case of the sum of the active and passive elements, the active elements, and the passive elements, respectively. A total of 2000 timesteps are used to calculate the total traveling distance. The plots show the averaged value over all the trials. In the left figure, the averaged total traveling distances for the circular, square, and triangular elements are 0.231
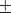
0.040 (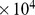), 0.335
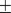
0.020 (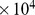), and 0.363
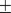
0.022 (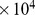), respectively. In the middle figure, they are 0.196
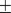
0.037 (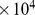), 0.258
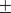
0.011 (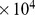), and 0.229
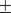
0.019 (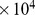), respectively. In the right figure, they are 0.0348
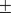
0.0066 (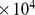), 0.0776
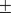
0.0193 (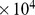), and 0.135
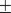
0.011 (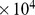), respectively. **B.** Comparisons of the averaged ratio of the traveling distances of the active elements (left) and the passive elements (right) in the total traveling distances of the circular, square, and triangular elements. The ratio is calculated for each trial and averaged over all the trials. In the active elements case, the averaged ratios for the circular, square, and triangular elements are 0.848
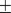
0.026, 0.770
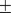
0.046, and 0.629
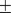
0.026, respectively. In the passive elements case, they are 0.152
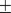
0.026, 0.230
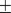
0.046, and 0.371
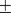
0.026, respectively. For both **A.** and **B.**, the error bars show the standard deviation. For each plot, asterisks indicate significant differences, 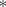: 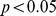, 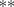: 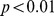, and 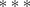: 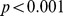.

In this section, we analyzed the total traveling distance (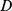) of elements for each shape. The traveling distance of an element can be considered to be a reflection of the amount of kinetic energy of the element. Therefore, by observing this, assuming that the energy flowing into the system is the same for each shape, we can estimate how the energy flow into the system is distributed to the kinetic energy of elements and how it dissipates. 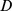 is defined as follows:
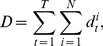
(1)

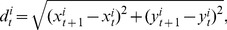
(2)where 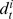 and 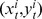 are the speed and position, respectively, of element 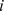 at timestep 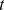. In addition, 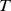 and 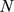 are 2000 and 12, respectively. Figure 5 A shows the total traveling distance for each shape averaged over five trials. In the sum of the total traveling distance for the active and passive elements (Fig. 5 A, left), we can see that, according to the change in shape to a circle, square, and triangle, the averaged traveling distance increases significantly, implying different energy dissipation characteristics for the various shapes. In addition, by comparing the total traveling distance of the active elements and the passive elements (Fig. 5 A, middle, and right, respectively), we can see that this increase, according to the change in shape, is mainly due to the total traveling distance of the passive elements. The plot in Fig. 5 B shows the averaged ratio of the traveling distance of the active and passive elements to the sum of the total traveling distance of both. As can be seen from the figure, according to the change of the shape to a circle, square, and triangle, the averaged ratio increases significantly in the passive elements. (Accordingly, the ratio of the total traveling distance of the active elements decreases). This tendency is consistent with the observation on the segregation phenomenon above; namely that in the circular element case, the passive elements are at the periphery of the container and do not move around very much. Conversely, in the triangular element case, they aggregate at the center of the container, constantly changing the formation of the cluster. Moreover, the square case shows intermediate behavior between the circular and triangular case. This result also suggests that the transformed kinetic energy is distributed more to the passive elements when the shape is changed to a circle, square, and triangle.

In the following sections, we aim to quantitatively characterize both the local interaction regimes of elements and the resulting global behavior in detail from a statistical point of view.

### Analysis of local interaction between elements

The focal measure in this section is the speed of each element at each timestep (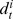). This variable would reflect the local dynamics of the elements and the interaction between elements, the friction in the water, the interaction between the wall, etc.

#### Probability distribution of 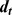


**Figure 6:**
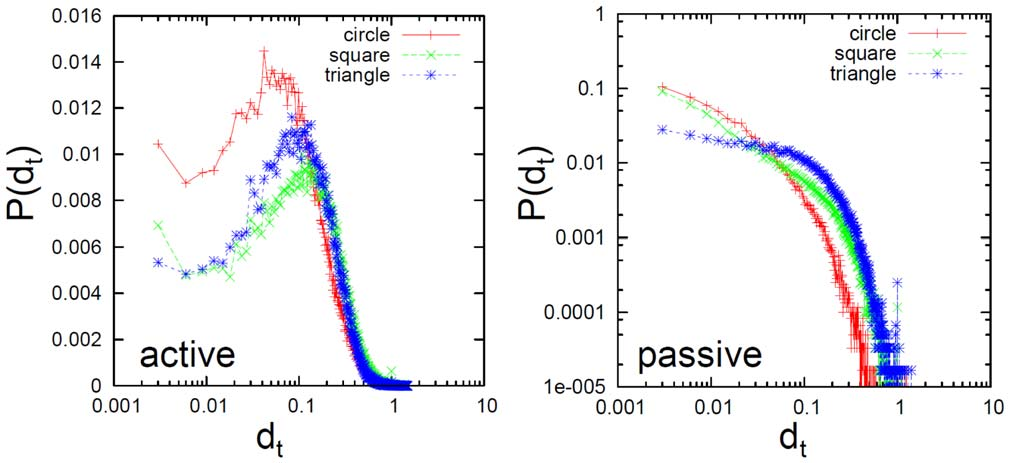
Comparisons of the probability distributions ( 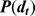
 **) as a function of** 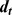 **.** Note that the plots of the active elements (left) are semi-log plots, while the plots of the passive elements (right) are log-log plots. For each plot of the probability distribution, we discretized the range (0, 1.5) into 500 bins and calculated the probability distribution, and then averaged over all trials. Note that, in the passive elements, the tails of the distributions for the square and the triangular cases seem to follow the power law. For the square case, the slope was −0.888 in the region of (0.003, 0.21), while for the triangular case, it was −0.345 in the region of (0.003, 0.09). The slopes were fitted by using the least-squares method.

Figure 6 shows the plots of the probability distribution of 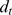 for each shape of the active and passive elements. For the active elements (Fig. 6 left diagram), we can see that the square case shows a peak at a higher value of 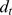 than for the circular and triangular cases. As we observed, the square elements did not show a clear segregation effect; accordingly, the active elements did not concentrate so much compared with the other two cases. This behavior is considered to be the cause of the peak in the square case. On the other hand, for the circular elements, we can see a peak at a relatively small value of 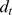. Also, the value of 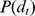 at the peak is larger than for the other shapes, which indicates that the active elements concentrate in the center of the container. In the passive elements (Fig. 6 right diagram), as the shape changes from the circle to the square to the triangle, it seems to show a power law distribution in the tail region and the slope of the tail region gradually becomes flat.

#### Associations between elements

In this section, we investigate associations between the elements by means of 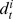. In statistical terms, two variables are associated if they are not independent. Mutual information is a general measure of association between two or more random variables, naturally encompassing both linear and nonlinear dependencies [33]. Let 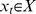 and 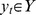 be a time series of the recorded variables at discrete time 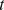. Furthermore, let 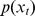, 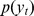, and 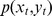 be the single and joint probabilities associated with variables 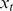 and 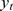. Mutual information measures statistical independence as:
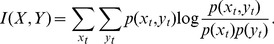
(3)For statistically independent distributions, 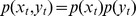 and 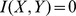. If there exist statistical dependencies, 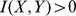. Given that entropy (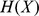) for variable 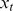 is expressed as:
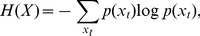
(4)the mutual information can be equivalently expressed by using entropy and conditional entropy as:
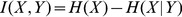
(5)

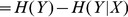
(6)

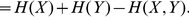
(7)It is obviously symmetrical over 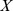 and 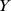, and 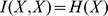. In our case, the behavior of the elements is intrinsically biased from the experimental setting; namely, the active elements vibrate randomly and the passive elements have no motivational force. In order to reduce this effect and to evaluate and compare the associations between the elements more clearly, it is desirable to introduce a normalized version of 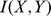 that ranges from 0 to 1. Several normalizations can be used based on the observation that 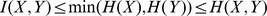 [34]–[37]. In this paper, we adopt normalized mutual information (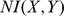) as follows [34], [36];
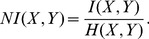
(8)Based on this measure, we compare the associations between the active elements (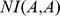), the active elements and the passive elements (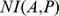), and the passive elements (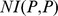) for each shape. Details on the calculation of 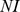 are found in Materials and Methods.

**Figure 7:**
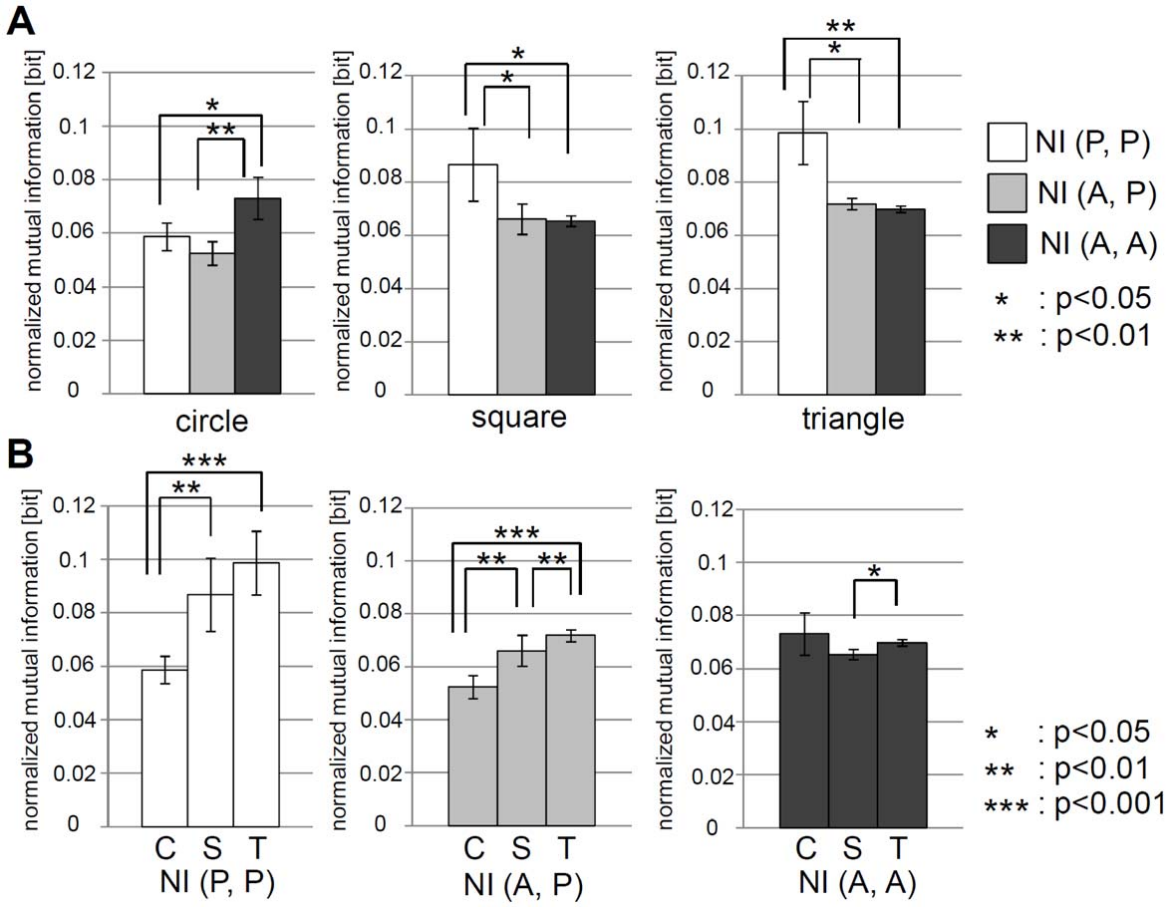
Comparisons of normalized mutual information ( 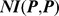
 **,** 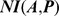 **, and** 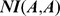 **).** **A.** Comparisons of normalized mutual information in the circular (left), square (middle), and triangular (right) cases. To calculate the normalized mutual information, we used the last 1200 timesteps for all the pairs of 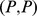 (15 pairs), 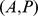 (36 pairs), and 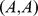 (15 pairs), in each trial. The plots show the averaged value over all the trials. The error bars show the standard deviation. In the circular case, 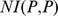, 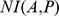, and 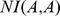 are 0.059
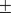
0.005 bits, 0.052
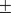
0.004 bits, and 0.073
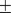
0.008 bits, respectively. In the square case, 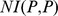, 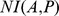, and 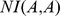 are 0.087
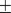
0.014 bits, 0.066
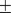
0.006 bits, and 0.065
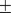
0.002 bits, respectively. In the triangular case, 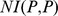, 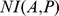, and 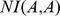 are 0.099
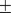
0.012 bits, 0.072
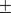
0.002 bits, and 0.070
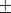
0.001 bits, respectively. **B.** Comparisons of the circular, square, and triangular cases for 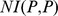, 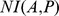, and 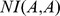. The same plots of **A.** are rearranged. For **A.** and **B.**, asterisks indicate significant differences, 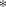: 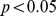, 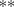: 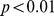, and 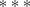: 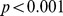.

Fig. 7 A shows the comparisons of 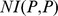, 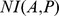, and 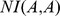 for each shape. For the circular elements, the association between the active elements shows the highest value, while for the square and triangular elements, the association between the passive elements shows the highest value. This result corresponds well with the visual observation of the elements' behavior. For the circular elements, as we observed, the active elements gathered in the center of the container. That is, as a result of gathering and collision, which affects the behavior of each other, 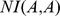 can be considered to be the highest. On the other hand, in the square and triangular elements, we observed that the passive elements bind each other and form clusters. As a result, 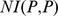 can be considered to be the highest. Furthermore, as can be seen in Fig. 7 B, for 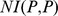 and 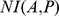, as the shape of the element changed to circle, square, and triangle in that order, the association tended to get higher. On the other hand, in 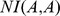, the incremental change of the value according to the transition of the shape was not clearly observed. Especially in 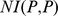, although we could not observe a significant difference between the square and triangular elements, we clearly observed a significant difference between the circular and square elements, and the circular and triangular elements. This result would be the outcome of the binding through capillary forces.

### Analysis of global behavior

The clusters observed were not always regular polygons, but also assume shapes that were incomplete as they slide along the side of the elements, changing the shape of the cluster. Therefore, it is hard to directly analyze the shape of the clusters. In this section, the diversity of clusters for components of different shape is quantitatively analyzed by introducing a statistical measure proposed by Balch [38].

Balch also considered this issue in a group of robots, stating that group diversity consisted of elements that could be characterized by the number of subgroups that are classified by some criteria, and the distribution of elements in each subgroup. In order to characterize the diversity of elements, Balch introduced a measure called *simple social entropy* (SSE) [38]. SSE (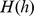) is expressed as follows:
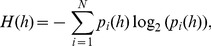
(9)where 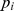 is the number of elements in the 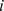
th subgroup (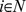) divided by the total number of elements (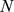). An element 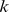 belongs to a subgroup if the distances between all the other elements belonging to the subgroup is within the length of 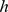 (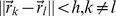; 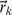 is the position of the 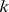
th element, and the element 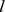 belongs to the subgroup). Note that the number of subgroups and the distribution of elements depends on 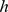, since the total number of elements are fixed. Basically, the value of SSE is higher if the number of subgroups is large and the elements are distributed to each subgroup equally. The value gets low if the number of subgroups is small and the distribution of elements to the subgroups is biased. That is, if all the elements are separately classified into each subgroup, the value of SSE is high, and if all the elements are in the same subgroup (group), the value is minimized at zero. As discussed in [38], SSE alone has a limitation as a diversity metric, since the value just reflects the diversity dependent on a specific 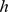. Also, it can indeed capture the difference between elements in the subgroups, but ignores the distribution of elements in the space. These limitations would be improved if the spatial structure of the system were considered, which is the distribution of elements in the classification space. In this regard, Balch proposed a measure called *hierarchic social entropy* (HSE) [38]. HSE (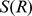) is expressed as follows:
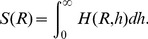
(10)where 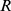 is the robotic system under evaluation, and 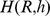 is the SSE for a specific 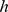 and 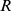. This measure reflects SSEs for all the possible groupings according to the parameter 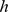. According to this measure, if the value is large, the diversity is said to be large; if the value is small, the diversity is said to be small.

In this paper, elements are already separated into two groups, namely the active elements and the passive elements from the experimental setting. Accepting this distinction, we analyze SSE and HSE for the active elements and the passive elements in each shape. We take a snapshot of the elements for each timestep. By varying the parameter 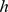, we calculate SSE for each 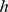 and HSE for each timestep. As the diameter of the container is 22.5 *cm*, varying 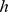 from 0 *cm* to 20 *cm* would be enough to calculate these measurements. We discretized the range from 0 *cm* to 20 *cm* into 500 segments, and varying the value of 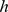 for each segment, we calculated SSE. HSE is calculated by summing up the SSE for all the segments. Details on the calculation of 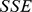 and 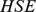 are found in Materials and Methods. By using these measures, we analyzed the behavioral diversity of circular, square, and triangular cases.

**Figure 8:**
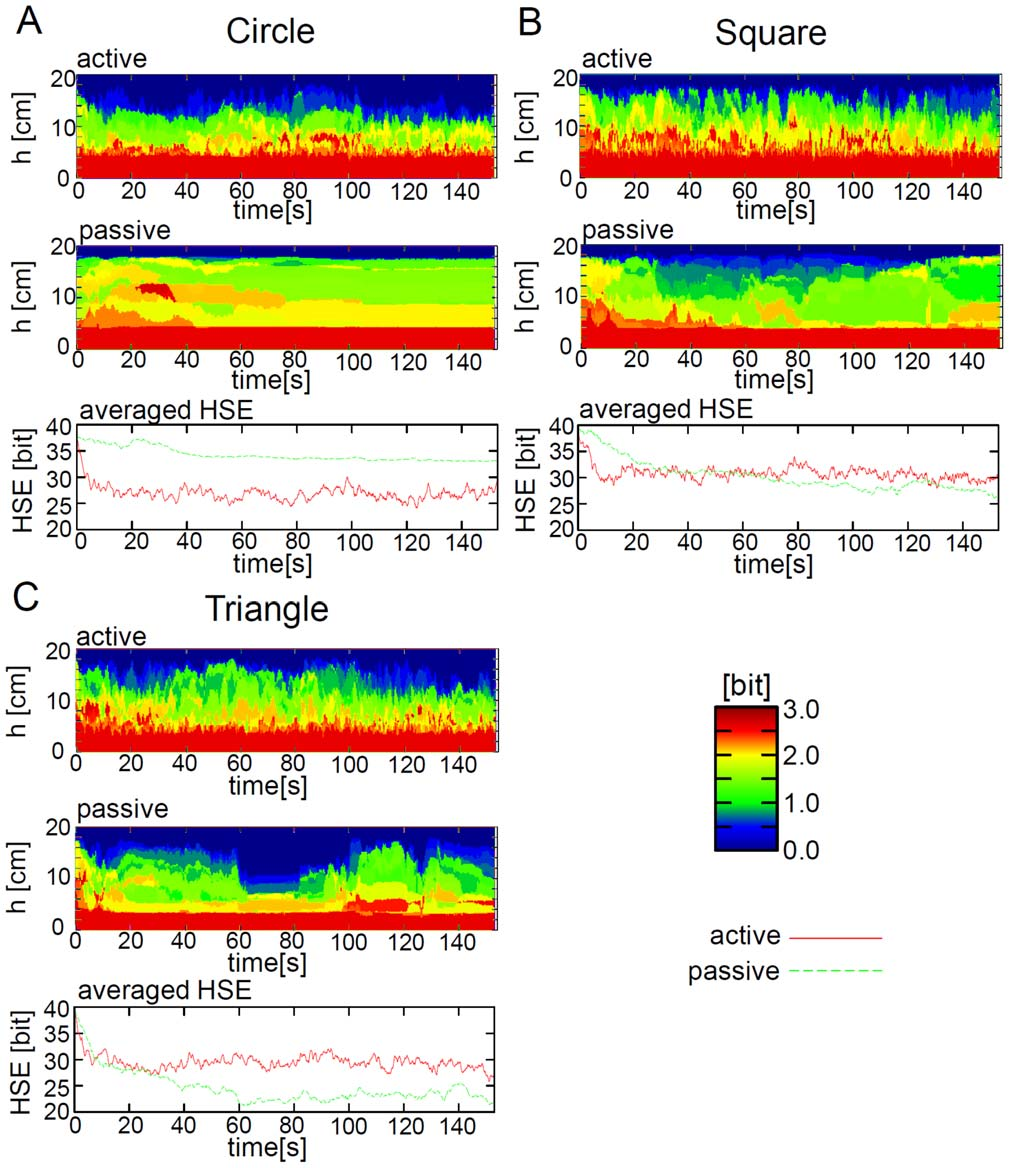
Typical examples of the SSE and the averaged HSE. **A**, **B**, and **C** show the circular, square, and triangular cases, respectively. For each shape, the upper and middle diagrams show the typical example of the SSE (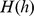) according to 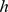 for each time for the active and passive elements, respectively. For each diagram, the brighter the color, the larger the value. The lower diagram shows the averaged HSE (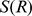) for the active and passive elements. The active and passive elements are overlaid in the same diagram. By calculating the HSE for the active and passive elements for each time and each trial, we averaged the values over all of the trials for each time.

We start by considering the case of the circular elements. The upper two diagrams of Fig. 8 A show the value of SSE according to the parameter 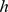 at each timestep for the active elements and the passive elements. The lower diagram of Fig. 8 A shows the plots of the dynamics of HSE averaged over 5 trials for the active and passive elements. As can be seen from the plots of SSE, in the active elements, when the value of 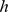 approaches 12–14 *cm*, the value of SSE is 0 bits; the value fluctuates over time. All the active elements can, therefore, be classified into one group with a relatively small 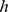. The active elements can be inferred to be clumped together through the action of constant fluctuation of their formation. On the other hand, for the passive elements the SSE value is not zero until the value of 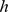 approaches 18 *cm*. This change seems to occur slowly, meaning that the passive elements are spreading out and do not change their configuration to a great degree in the container. These features are clearly expressed in the plots of the averaged HSE. Just after the start of the experiment, the HSE of the active elements drops suddenly from around 38 bits to 27 bits, and retains its value. The HSE for the passive elements does not change much from its initial value of 38 bits; this value is maintained to the end of the experiment. With these results, it can be said that for the case of the circular elements, the passive elements retain lower diversity than the passive elements, which seem to reflect the clustering of the active elements.

Now, let us examine the case of the square elements (Fig. 8 B). The value of the SSE in the active elements case does not reach zero until the value of 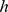 attains a relatively higher value (around 14 to 18 *cm*) in comparison with the value for the circular elements; this value fluctuates over time (Fig. 8 B, the upper diagram). For passive elements, we can see that the value fluctuates to a greater degree over time than it does for the active elements, and the SSE value almost reaches zero with a relatively lower value of 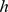 than was the case for the circular shapes (Fig. 8 B, the middle diagram). The active elements, therefore, are most likely further from each other in the container, while the passive elements are clustering more than in the case of the passive circular elements. From the HSE results, it can be seen that both the active and passive elements show almost the same value - about 30 bits - which is different from that for the circular elements (Fig. 8 B, the lower diagram). The diversity for the passive elements is lower, and the diversity for the active elements is high, in comparison to the circular elements.

Figure 8 C shows the plots for the triangular elements. As for the SSE in the active elements case, the value of the SSE shows zero at a similar value of 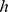 as for the square elements (around 14 to 18 *cm*). For the passive elements, the SSE is zero at a generally low 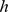 (around 16 *cm*, at its highest level). Moreover, even for the low 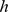 values, the value of 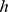 becomes extremely low and extremely high intermittently. This suggests that the active elements spread wider in the container, while the passive elements clump together a great deal during a particular point in time. From the results of HSE, it can be seen that the passive elements maintain an extremely low value of between 20 bits to 25 bits, while the active elements constantly maintain a value of around 30 bits. In other words, the diversity of the passive elements is reduced, and the diversity of the active elements increases, in comparison with the circular elements.

**Figure 9:**
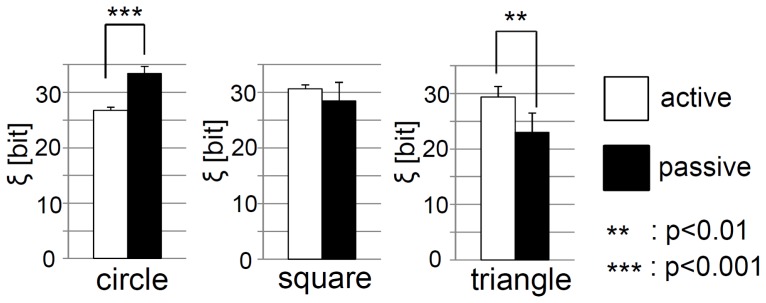
The averaged 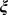
 **of the active and passive elements for each shape.** To obtain the averaged value of 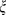, we calculated 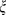 for each trial using the last 1200 timesteps and averaged 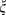 over all the trials. The error bars show the standard deviation. Asterisks indicate significant differences, 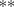: 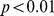, 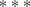: 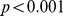. In the circular case, the averaged values of 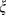 for the active and passive elements are 26.72
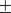
0.64 bits and 33.42
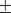
1.28 bits, respectively. In the square case, the averaged values of 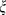 for the active and passive elements are 30.62
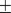
0.77 bits and 28.49
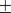
3.35 bits, respectively. In the triangular case, the averaged values of 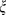 for the active and passive elements are 29.33
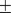
1.97 bits and 22.98
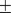
3.51 bits, respectively.

To summarize the HSE results for the circular, square, and triangular elements, we begin by comparing each case with the introduction of the measure 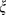, which is the averaged HSE over the specific time region expressed as follows:
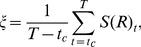
(11)where 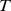 is the last timestep and 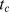 is the first timestep of the data used in a single trial. We used the last 1200 timesteps to calculate 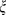 for each trial. Fig. 9 shows the averaged 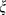 for the purpose of comparing the active and passive elements among the circular, square, and triangular elements. We observed a significant difference between the HSE of the active and the passive elements for the circular and triangular cases. It should be noted that the large or small order of the averaged 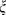 between the active and the passive elements is clearly reversed for the circular and triangular elements. These results correspond well with the behaviors of the active and the passive elements that were observed.

**Figure 10:**
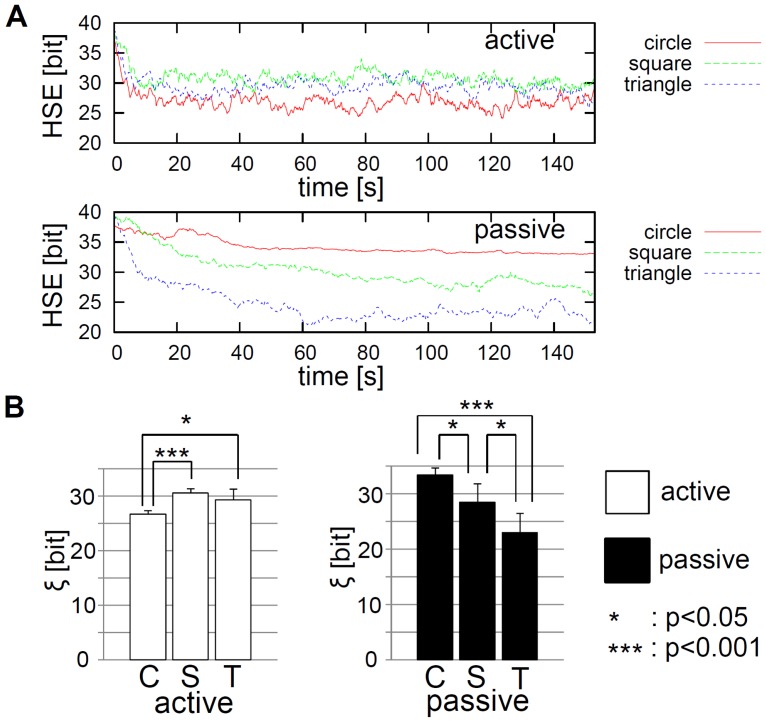
Comparisons of the averaged HSE and the averaged 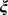
 **.** **A.** Comparisons of the averaged HSE between the circular, square, and triangular cases for the active elements (the upper figure) and the passive elements (the lower figure). They are the same plot lines shown in the lower line of Fig. 8 **A.**, Fig. 8 **B.**, and Fig. 8 **C.**, but rearranged and overlaid into two plot lines; namely, the plot lines for the active and the passive elements. **B.** Comparisons of the averaged 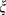 between the circular, square, and triangular cases for the active elements and the passive elements. They are from the same plot shown in Fig. 9, but rearranged into bar graphs, one showing the active elements, one showing the passive elements. Asterisks indicate significant differences, 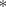: 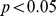, 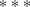: 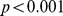.

The HSE for the active elements and passive elements is overlaid in the plot lines shown in Fig. 10 A. Greater differences in HSE are observable between the three different types of elements for passive elements in comparison to active elements. The value of HSE for the passive elements is highest for the circular elements, and then lower for the square and triangular elements, indicating a greater degree of clustering. Furthermore, comparisons of the averaged 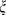 are shown for each morphology between the active and passive elements (Fig. 10 B). For the active elements, the value of 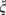 for square and triangular elements is higher than that for the circular elements; this difference is significant. This indicates a higher degree of clustering among the active elements for the circular shapes. As per the observation initially made, the difference in shape for the three elements clearly influences the formation of clusters, and whether clusters are more likely to form among active or passive elements.

## Summary and Discussion

In this paper, we have quantitatively characterized the difference of the segregation patterns among circular, square, and triangular cases. In the circular case, we observed that the active elements tended to gather in the center of the container, while the passive elements were located at the periphery of the container. In the square case, although we observed clustering of the passive elements, the segregation effect was less prominent. In the triangular case, the clustering of the passive elements was more enhanced compared to the square case, and they were frequently observed near the center of the container, while the active elements were observed at the periphery of the container. It is emphasized that with the change of shape from circular to triangular, the location resulting from the segregation of the active and passive elements is clearly inverted. Furthermore, focusing on the speed of the elements, we characterized the association between the elements by means of normalized mutual information. As a result, supporting our observation, in the circular case, the association of the active elements was high, while in the square and triangular cases, that of the passive elements was high. These results are considered to characterize the local interaction regime between the elements; namely, in the circular case, the active elements gather together in the center of the container and affect each other, while in the square and triangular cases, it was the passive elements that formed clusters. We also characterized the global behavior of elements for each shape by means of SSE and HSE, and obtained the quantitative results that support our observation. That is, in the circular case, the diversity of the active elements is lower than that of the passive elements, while in the triangular case, the tendency was reversed. In the square case, we could not see a significant difference between the active and passive elements. On the other hand, we observed that the diversity of the passive elements decreases according to the change of the shape from circle to square. These are also considered to be results of the formation of clusters for each shape.

A potential explanation for the segregation effect we observed may be a mechanism similar to the one leading to so-called entropic forces or depletion forces [39], a well-known effect in statistical physics and of great importance in biology (see, for example, [40]). Entropic forces are not “real” forces in the sense that they can be explained by a potential, but rather by a statistical effect. A prominent example is given where large hard spheres are immersed in a fluid or dense gas of small hard spheres [40]–[42]. The large spheres will almost always be found either clustered together and/or in close proximity to the wall of the container. The effect, to be explained below, creates a situation that looks similar to a scenario where there is an actual attraction between two large spheres and between a large sphere and a wall, hence the use of the term “force”. In order to understand this effect, note that each large sphere defines a region in which the centers of the small spheres can never enter (this region is larger than the actual radius of the large sphere; it is also spherical and has a radius equal to the sum of the large and small spheres). If the large spheres are in close proximity, these forbidden regions overlap, effectively leading to an increase of the volume available to the small spheres. For entropic reasons, as long as there are thermal fluctuations in the system, the large spheres tend to stick together. A very similar line of argument explains why large spheres stick to the wall. It is important to note that despite the appearance that the large spheres attract each other, the effect is in fact caused by the repulsion of the large and small spheres. We speculate that such a form of entropic force also plays a role in the observed segregation effect, but we emphasize that in a system involving floating components, there is also another form of interaction not dependent on (random) actuation, namely capillary forces.

In order to explain the observed segregation behavior in more detail, we first focus on the passive elements, namely the behavior of the clusters formed by them. These bindings between the passive elements are supposed to be caused by the capillary forces acting on them. Several studies exist that show self-assembly by exploiting capillary forces in the water-floating elements [9], [10], [14], [15], [17]–[19], [43]–[46]. In our experimental setting, since the footprints, as well as the mass of the elements, are set to be equal in all the different shapes, the number of sides in each shape is supposed to be regulating the strength of this force. In the triangular case, the length of the side is the largest, so its binding strength, which sustains the other binding elements, is supposed to be the largest. On the other hand, in the circular case, since the elements can contact each other only at one point in principle, the strength of the capillary forces acting on them is the lowest. From this speculation, we can understand the difference in the stability of the clusters among different shapes.

The force created by the active elements may be similar to the entropic force explained above. In that way, for the circular elements, since the passive elements do not form clusters, the active elements push the passive elements to the periphery of the container in order to maximize the free area available. This results in the gathering of the active elements in the center of the container. For the square and triangular elements, since the passive elements can form clusters due to capillary forces, the active elements can potentially have two ways to maximize their available free area; namely, make the passive elements gather together or be pushed toward the periphery of the container. In our experiment, due to the stability of the clusters, the clusters of the square elements were not stable enough to maintain their formation against the collision of the active elements. As a result, clear segregation was not observed. For the triangular elements, the clusters of passive elements were more stable due to the capillary forces, and this ensured that the active elements would be pushed to the periphery more often. In summary, the combination of the capillary forces on the passive elements and the entropic force caused by the active elements provides an explanation for the reverse segregation pattern that occurred in the following order: circle, square, and triangle.

The detailed analysis of the mechanism should be presented in future work. One possible direction would be to investigate to what factor of segregation the energy flowing into the system is distributed, by focusing on the amount of energy dissipation. In addition, one way to investigate our speculation experimentally would be to vary the control parameters of the system and observe how the final outcome of the segregation changes. As explained above, the entropic force and capillary force play an important role in our hypothesis; entropic force would be related to the collision rate of the elements. Considering this point, by varying the control parameters of the system (such as the voltage applied to the system or the number of elements, which would change the collision rate of elements) and observing the change in the efficiency of the segregation, it would be possible to further investigate the role of entropic force in the segregation phenomenon. Also, the experiment, with the addition of small quantities of detergent to fluid, may help reveal the role of capillary force.

Segregation can be viewed from a physical perspective and, especially in the dynamic case, constitutes a non-trivial collective phenomenon. But segregation can also be exploited as a fundamental process in technology. We sketch an initial scenario: assume a large number of agents, all of them performing energy-consuming tasks. As long as they are active, they should reside in the “working area,” and as soon as they stop being functional (e.g. by emptying their batteries), they should move into a “maintenance area”. Either these agents have a possibility to notice when they are running out of fuel and can travel back to a recharging station, or a centrally organized pick-and-place mechanism has to be provided. The latter adds an additional level of sophistication to the system; the former requires the agents to be capable of predicting how much energy they need to return to a charging station. In partially unknown settings, this prediction may well be difficult or unreliable. A segregation process, in contrast, in which deactivated agents are transported automatically and in a self-organized manner, out of the working region into the maintenance area, avoids these two problems. It has to be noted that agents not only devoid of energy, but out of function, are also autonomously transported to the border of the system, thereby constituting a self-healing process.

Another potential scenario for applications is given in which production processes requiring different steps performed by modules, if brought into close proximity, would interfere with each other. Assuming that the (pre-) products are allowed to move freely, segregation leads to a highly adaptive form of self-organized compartmentalization without the need to construct actual physical barriers (which, in turn, may hinder the transport of products). Further, the segregation effect might be used by industry to avoid the Brazil nut effect [11] through better understanding of the mechanisms taking place. By forcing a module out of the position it might occupy due to its size, a more heterogeneous distribution of modules can be achieved.

## Materials and Methods

### Experimental Conditions

**Figure 11:**
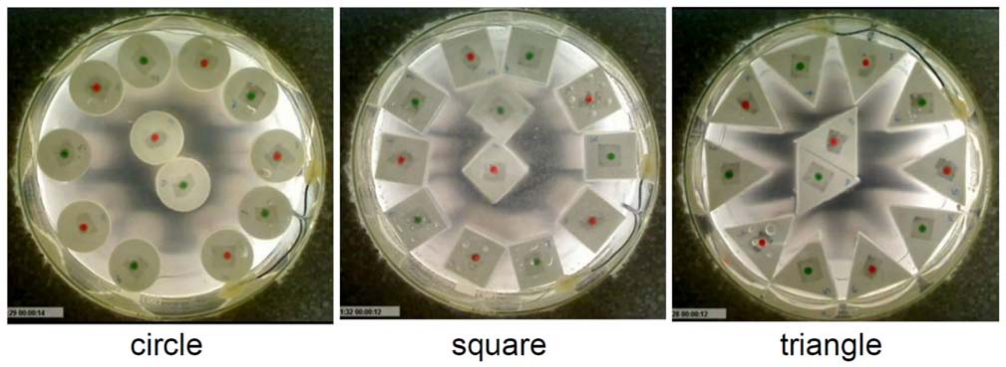
Initial configurations for the circular, square, and triangular cases. Note that the elements' centers are colored red and green to indicate the active and passive elements, respectively.

The experimental platform used in this paper is based on the platform developed in [27]–[32], but is not equipped with magnets. The active elements are equipped with vibration motors (FM34F, 12000–14000 *rpm* (2.5–3.5 *V*)), a pantograph, and electrodes, which can derive electricity via the pantograph from the ceiling (Fig. 1). When a voltage is applied to the ceiling plate (7 *V* (E in Figure 1)), the current runs through the pantograph to the vibration motor, returning to the ground via the electrode immersed in the conductive water (NaCl solution, 83.3 *g/l*). Passive elements have no motor. Due to this setup, active elements receive equivalent current flow in cooperation with the following chemical reactions.
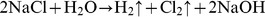
(12)The concentration of the NaCl solution is sufficient to sustain constant current flow during the experiment. To avoid chemical deposition onto the electrode, platinum is used for the material. The initial configuration of the elements was set as shown in Fig. 11 (0 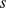), in which 5 active and 5 passive elements were alternately aligned in a circular form in a symmetrical fashion, and the remaining elements (1 active and 1 passive) were placed near the center to guarantee the maintenance of symmetry. Our aim in this paper is to assess how the shape of elements alters the segregation phenomenon. Therefore, we used a fixed initial configuration of elements throughout all the experiments and among the different shapes. A camera was set under the transparent container to observe the behavior from the bottom. For the results, in which we measure the positions of the elements, we track the center of the elements using a *Tracker Video Analysis and Modeling Tool* [47].

### Normalized Mutual Information

**Figure 12:**
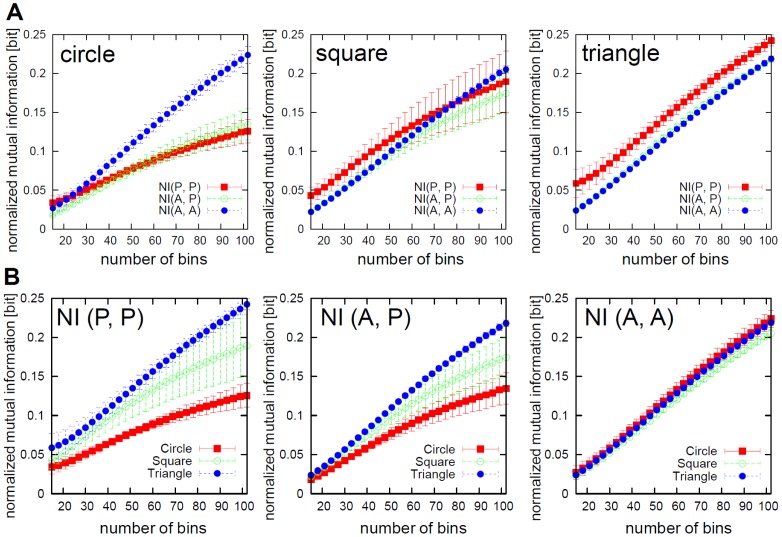
Comparisons of normalized mutual information according to the number of bins. **A.** Comparisons of normalized mutual information (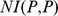, 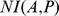, and 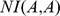) according to the number of bins in the circular (left), square (middle), and triangular (right) cases. **B.** Comparisons of the circular, square and triangular cases for 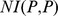, 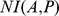, and 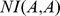. The same plots of **A.** are rearranged.

In order to extract the property of the time series of 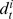 shown in Fig. 6, we used the data equipped with the common logarithm, 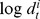, to calculate the normalized mutual information. The time series of 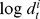 ranging from −2 to 0 were discretized to several bins, and single probabilities and joint probabilities used to calculate the normalized mutual information were approximated by means of histograms. The number of bins was set to 36 in the result. Fig. 12 shows the results of the normalized mutual information according to the number of bins. We should note that the relative large or small orderings of 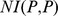, 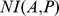, and 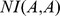 for the circular and square cases changed according to the specific number of bins (Fig. 12 A); namely, when they were smaller than 27 (Fig. 12 A, the left diagram), and when they were larger than 79 (Fig. 12 A, the middle diagram), respectively. Otherwise, we can confirm that the tendency we observed in this paper was qualitatively robust with respect to the specific choice of bins.

In each trial, by using the last 1200 timesteps, we calculated 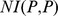, 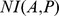, and 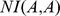 for each pair of the elements and averaged over all the pairs of 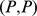 (15 pairs), 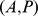 (36 pairs), and 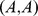 (15 pairs), respectively. For 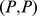 and 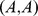, the pairs of the same elements were excluded. Furthermore, 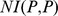, 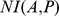, and 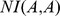 are averaged over all 5 trials and used as a result (standard deviations shown in Fig. 7 and Fig. 12 were derived during this process).

### Simple Social Entropy and Hierarchic Social Entropy

**Figure 13:**
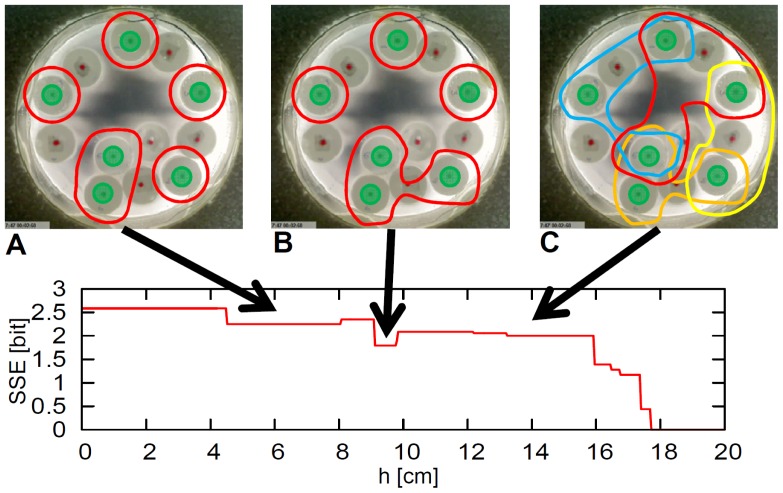
Schematics explaining how to calculate the SSE and the HSE. The figure shows the example from the certain configuration (certain timestep) of the passive elements in the circular case. **A.** The elements are grouped with 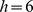 into five subgroups. **B.** The elements are grouped with 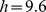 into four subgroups. **C.** The elements are grouped with 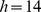 into four overlapped subgroups.

We explain how we calculated SSE and HSE in this paper. By taking a snapshot of the elements for each timestep, we varied parameter 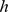 and calculated SSE for each 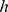 and HSE for each timestep. As shown in Fig. 13 A, when 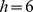, we have five subgroups. Four of them consist of a single element (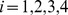), and the other one consists of two elements (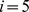). So . When 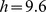, we have four subgroups (Fig. 13 B). Three of them consist of a single element (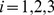), and one of them consists of three elements (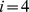). So . When 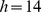, we can find four overlapped subgroups (Fig. 13 C). Three of them consist of three elements (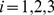), which are grouped with red, orange, and blue lines; the other one, which is indicated with a yellow line, consists of two elements (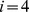). So, . By summing up all the SSE (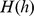) according to 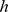, we obtain the value of the HSE.

## Supporting Information

Video S1 **Video showing typical segregation phenomena for circular, square, and triangular case.** For each shape, active and passive elements are marked in red and green, respectively. The speed of the video is set eight times faster than the actual running speed.
(WMV)
